# Evidence for two catalytic centers and Mn^2+^ as physiological cofactor of soluble guanylyl cyclase

**DOI:** 10.1186/1471-2210-11-S1-P7

**Published:** 2011-08-01

**Authors:** Kerstin Yvonne Beste, Johannes-Peter Stasch, Volkhard Kaever, Roland Seifert

**Affiliations:** 1 Institute of Pharmacology, Hannover Medical School, Hannover, Germany; 2 Institute of Cardiovascular Research, Bayer HealthCare, Wuppertal, Germany

## Background

Soluble guanylyl cyclase (sGC) constitutes a family of enzymes that catalyses the cyclization of guanosine 5’-triphosphate (GTP) to guanosine 3’,5’-cyclic monophosphate (cGMP). The heterodimeric hemoprotein is activated by nitric oxide and mediates a wide range of physiological effects like regulation of blood pressure and neuronal cell development. sGC is an important target for the treatment of cardiovascular diseases.

In addition to the biosynthesis of cGMP, we recently showed that *invitro* and in presence of Mn^2+^ ions sGC also generates the cyclic purine nucleotides adenosine 3’,5’-cyclic monophosphate (cAMP), inosine 3’,5’-cyclic monophosphate (cIMP), and xanthosine 3’,5’-cyclic monophosphate (cXMP)[1]. For all purine nucleotides, a second low-affinity site was described[1]. Moreover, sGC shows a pyrimidinylyl cyclase activity for uridine 3’,5’-cyclic monophosphate (cUMP) and cytosine 3’,5’-cyclic monophosphate (cCMP). In this case, no second binding site could be identified[1]. Lastly, no formation of the cyclic desoxyribonucleotide thymidine 3’,5’-cyclic monophosphate (cTMP) was detected[1].

## Materials and methods

For *in vitro* assays and determination of Michaelis-Menten kinetic parameters highly purified recombinant sGC from rat (α_1_β_1_) was activated by sodium nitroprusside (100 µM). sGC (0.1-20 ng/tube) was incubated at 37°C with 3 mM MnCl_2_ or MgCl_2_ and various concentrations of nucleoside 5’-triphosphates (NTPs). Samples were stopped by heating at 95°C. Concentrations of cyclic nucleotides were determined by HPLC-MS/MS.

The *in vivo* assays were performed using transiently α_1_β_1_-transfected HEK 293 and endogenously sGC-expressing rat fetal lung fibroblast RFL-6 cells[2]. Cells were stimulated with SNP (100 µM) with or without phosphodiesterase inhibitor 3-isobutyl-1-methylxanthine (IBMX, 100 µM). After defined times medium was removed and cell metabolism was stopped by addition of organic solvent and by heating at 98°C. After centrifugation clear supernatant was taken for quantitation of cyclic nucleotides by HPLC-MS/MS.

## Results

Analyzing *in vitro* substrate specificity in presence of Mg^2+^ revealed that cyclase activity of sGC is limited to purine nucleotides. Beside cGMP, cAMP, cIMP, and cXMP, but no cUMP and cCMP were generated. Additionally, in contrast to Mn^2+^ as cofactor, kinetics could be described completely using a model of a single binding site, suggesting that when Mg^2+^ ions are present only one high-affinity binding site is active.

**Figure 1:**
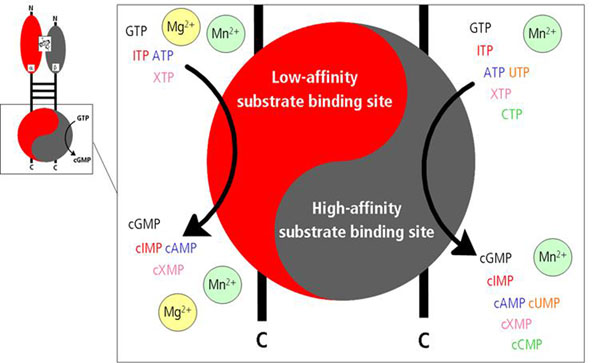
Model of two catalytic active binding sites in sGC

However, studying transiently sGC-transfected HEK293 and endogenously sGC-expressing RFL-6 cells after SNP-stimulation we could detect a time-dependent accumulation of cGMP and cAMP as well as a generation of cCMP and cUMP. cXMP, cIMP and cTMP could not been detected. The addition of IBMX could only inhibit degradation of cGMP and cAMP while concentrations of cCMP and cUMP were only marginally influenced. These data indicate that Mn^2+^ rather than Mg^2+^ is the relevant divalent cation for sGC. Moreover, cCMP and cUMP appear to be hydrolysis-resistant in HEK293 and RFL-6 cells. Figure 1.

## Conclusion

1. In the presence of Mg^2+^, sGC exhibits one catalytic center but in the presence of Mn^2+^, there are two catalytic centers.

2. Furthermore, in intact cells, NO not only stimulates cGMP production, but also cAMP, cCMP and cUMP synthesis.

3. These data indicate that *in vivo*, Mn^2+^ rather than Mg^2+^ may be the relevant divalent cation for sGC.
